# Treatment-Seeking Behaviour and Social Health Insurance in Africa: The Case of Ghana Under the National Health Insurance Scheme

**DOI:** 10.5539/gjhs.v7n1p296

**Published:** 2014-10-27

**Authors:** Ama P. Fenny, Felix A. Asante, Ulrika Enemark, Kristian S. Hansen

**Affiliations:** 1 Economics Division, ISSER, University of Ghana, P.O. Box LG 74, Legon, LG74, Accra, Ghana; 2 Department of Public Health, Aarhus University, Vennelyst Boulevard 6, 8000 Århus C, Denmark; 3 Department of Global Health and Development, Faculty of Public Health and Policy, London School of Hygiene and Tropical Medicine, 15-17 Tavistock Place, WC1H 9SH, London, UK

**Keywords:** health insurance, health care utilisation, multinomial logit model, household survey, Ghana

## Abstract

Health insurance is attracting more and more attention as a means for improving health care utilization and protecting households against impoverishment from out-of-pocket expenditures. Currently about 52 percent of the resources for financing health care services come from out of pocket sources or user fees in Africa. Therefore, Ghana serves as in interesting case study as it has successfully expanded coverage of the National Health Insurance Scheme (NHIS). The study aims to establish the treatment-seeking behaviour of households in Ghana under the NHI policy.

The study relies on household data collected from three districts in Ghana covering the 3 ecological zones namely the coastal, forest and savannah. Out of the 1013 who sought care in the previous 4 weeks, 60% were insured and 71% of them sought care from a formal health facility. The results from the multinomial logit estimations show that health insurance and travel time to health facility are significant determinants of health care demand. Overall, compared to the uninsured, the insured are more likely to choose formal health facilities than informal care including self-medication when ill. We discuss the implications of these results as the concept of the NHIS grows widely in Ghana and serves as a good model for other African countries.

## 1. Introduction

Despite efforts to improve the provision of health services, many low- and middle-income countries are still far from achieving universal health coverage (World Health Report, 2010). The financing of healthcare in Africa remains a patchwork of meagre public spending, heavy reliance on foreign donors and a large dependence on out-of-pocket contributions and user fees that place the greatest burden on the poorest members of society (World Health Report, 2010). The debate on health sector reform is the need to move away from excessive reliance on direct out-of-pocket payment to pre-payment and risk-sharing (Claeson et al., 2001). A number of countries are trying to establish or broaden social insurance programmes to improve access to health care of their citizens (Wagstaff, 2009).

Social health insurance (SHI) is described as a government-sponsored health care financing mechanism that is driven by social solidarity values and based on pooling health risks of those enrolled (Wagstaff, 2009). Several low- and middle-income countries, including the Philippines, Thailand and Viet Nam, are establishing SHI (Spaan et al., 2012). In Sub Saharan Africa, countries such as Senegal, Rwanda, Tanzania, Kenya and Nigeria have implemented several variations of social health insurance schemes (Witter & Garshong, 2009; Nyantaki, 2009; Gobah et al. 2011). An overview of the scope and origin of SHI in low- and middle-income countries concludes that the picture in Africa and Asia is very patchy, with large differences in population coverage, services covered and costs achieved (Soors et al., 2010). Ghana, Senegal and Rwanda are among leading countries that have experimented with the idea of Community–based Health Insurance Schemes (CBHISs) as a national health program in Africa (Jutting, 2003).

Ghana took a bold initiative to reform the health sector by introducing a National Health Insurance Scheme in 2005. The main objective of the scheme is to promote access to quality health care services (Ghana Ministry of Health, 2004a). Such access could constitute a substantial contribution to breaking the cycle of poverty and poor health. The NHIS in Ghana is an interesting case study for a number of reasons. The first and foremost is that Ghana has scaled up the coverage of the NHIS from about 2.5 million active members in 2006 to about 8.9 million by 2012, representing 35 percent of the total population (NHIA, 2013). Secondly, this has attracted so much interest that other countries in the sub-region are considering the Ghanaian model as an alternative vehicle for health sector financing. Countries such as South Africa and Tanzania have been less successful in transitioning to national health insurance schemes (McIntyre et al., 2008).

A review by the Ghana Health Service (2008) shows that since the start of the National Health Insurance Scheme (NHIS) in 2005, overall Out Patient Department (OPD) cases have shown a marked increase, suggesting that the NHI policy has led to an increase in health service usage. Some studies in the recent past, using pilot cases in one or two districts in Ghana have also pointed to the fact that rural health insurance could serve as a viable alternative to user fees by removing barriers to utilization of health care (Atim and Sock, 2000; Arhin, 1995; Osei-Akoto, 2004). Some strong critiques of the NHIS program argue that the scheme has the potential to further alienate the extreme poor from utilising health services (Apoya & Marriott, 2011). Apart from the high costs of premiums, there are other direct and indirect costs that needs to be considered; which make it difficult for poor people to fully utilize the scheme. Expenses such as transport, prescription drugs, others including the opportunity cost of time especially for informal workers. Various factors including socio-demographic characteristics, family and individual resources and health condition influence treatment-seeking behaviour (Sahn et al., 2003; Lindelow, 2005; Anyanwu, 2007; Gwatkin et al., 2007; Amin et al., 2010; Sato, 2012).

This study attempts to contribute to this debate by providing some evidence on the key determinants of utilisation paying close attention to one of the factors hypothesized to affect patient demand, namely health insurance status. The research questions addressed in this paper are: has the NHIS in Ghana assisted households to change their behaviour towards formal healthcare utilization? What are the determinants of utilisation: how do the poor fare in both instances? A unique feature of this study is the addition of an interaction term between insurance status and wealth status in order to understand the extent to which wealth influences the effect of health insurance on choice of care.

Ideally these research questions could have been addressed with little or no bias if the data were generated from a fully randomised experiment. In our case, the data was generated from a field survey so there is no guarantee that membership to the NHIS is entirely random. There are potential selection biases generated from the following: individuals with pre-existing condition may self-select into the NHIS raising the classic problem of moral hazard. Richer households will enrol because it is cheaper for them to. Also, the premium could be too high for the poor including other unobserved factors. All these factors could lead to biases on any estimator that attempts to establish causality stemming from NHIS membership outcomes.

Relying on the behavioural model and subsequent modifications (Andersen, 1995; Andersen & Newman, 2005), this paper assesses the determinants of choice of care in Ghana bearing in mind the endogeniety issue raised above. These results can therefore help policy makers to understand patient treatment-seeking behaviour, and subsequently provide information for developing future health policies.

The next section presents an overview of the health sector and attributes of health providers as well as the structure of the health insurance system in Ghana. Section three presents a review of health seeking behaviour specifically looking at the link between health insurance and provider choice. Section four describes the estimation methods and empirical specification. Section five presents the results of the empirical analysis with the conclusion and policy implication presented in section six and seven respectively.

## 2. Healthcare and Health Insurance in Ghana

### 2.1 Structure of Formal Health Facilities

**Figure 1:**
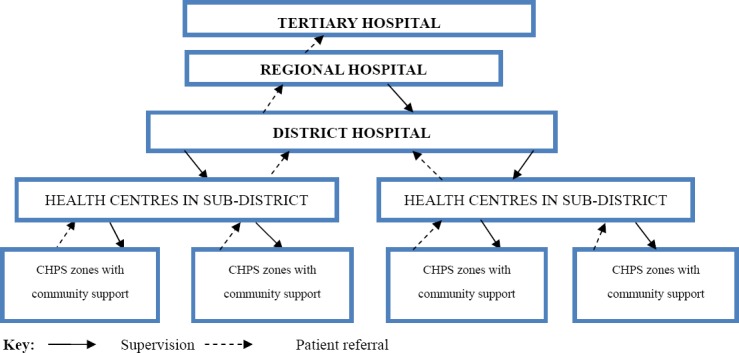
Organizational Structure of the Public Healthcare System Source: MOH, 2009

Health care delivery in Ghana is provided by both the public and private (private-for-profit and private-not-for-profit) sectors, with the public sector organized according to hierarchy with national (teaching hospitals) at the apex, followed by regional (regional hospitals), district (district hospitals), sub-district (health centres) and community levels (CHPS). Community-based Health Planning and Services (CHPS) is a programme for transforming clinic based primary health care to community-based health services. Figure 1 shows the hierarchical organisation of formal health facilities in Ghana.

Sub-district (health centres) and community levels (CHPS) provide primary care, with district and regional hospitals providing secondary health care as well as primary health care. The tertiary services including specialised clinical care are provided at the teaching hospitals. District hospitals are staffed with one or more qualified medical doctors, nurses, pharmacists, laboratory technicians, auxiliary nurses and other support personnel. Health centers are manned by a medical assistant or a nurse. This hierarchical structure incorporates a referral system from lower levels to the level immediately above them. However, weak health systems at the primary level and a weak referral system mean that many individuals visit secondary and tertiary levels for outpatient care.

### 2.2 Rationale for the National Health Insurance Scheme in Ghana

Health care financing in Ghana has gone through many dynamics, from free health care at the eve of independence, introduction of the nominal fee in the 1970s and the 1980s full cost recovery, popularly known as the ‘Cash and Carry’ system. Recognizing that direct out-of-pocket payment limited access to health care, the Government of Ghana declared its intention to abolish the system, and began exploring the feasibility of introducing a national health insurance scheme to be managed at the district level.

The National Health Insurance Act, 2003 (Act 650) established the National Health Insurance Scheme (NHIS) with the aim of increasing access to health care and improving the quality of basic health care services for all citizens, especially the poor and vulnerable. The law establishing the scheme allows for the concurrent operation of District-Wide (Public) Mutual Health Insurance schemes, Private Mutual Health Insurance schemes and Private Commercial Health Insurance schemes. The defined benefit package under the scheme includes inpatient hospital care, outpatient care at primary and secondary levels, and emergency and transfer services. Premium contribution is charged each client and is renewable on yearly basis. Members can have access to services 6 months after registration to curb adverse selection. The NHIS has an exemption policy in place to ensure that the poor and vulnerable groups in the society have access to healthcare; the exempted groups include the poor, children under the age of 18 years and the elderly (70 years and above).

Under the NHIS, all public health facilities are automatically accredited to the NHIS. Private health facilities on the other hand have to apply to the National Health Insurance Authority (NHIA) for accreditation to participate in the scheme. The accreditation criteria often compel some private facilities to improve the range of services and quality of services. Some of the areas scrutinized include the number of qualified health personnel, availability and quality of utilities such as regular supply of water and electricity. Patients with valid NHIS cards may to choose where to go for health care (public or private accredited health providers) once they are covered under the scheme in the district.

## 3. Health Seeking Behaviour: Linking Health Insurance and Choice of Provider

Behavioural responses to health utilization have been studied in various settings across the globe. A range of patient characteristics determines whether patients are willing and able to make treatment choices. Some of these choices may also be influenced by social and cultural factors (Sahn et al. 2003; Lindelow, 2005; Amin et al., 2010, Kaija and Okwi, 2010). One of the frequently used frameworks for analyzing factors that are linked to patients’ choice of care is the Andersen–Newman framework (Andersen 1995; Andersen and Newman, 2005). Here an individual’s access to healthcare is based on three facets (predisposing, enabling and needs factors). Predisposing factors include socio-demographic characteristics (age, gender, education); enabling factors include individual, family and community resources which can include income, costs of care, health insurance, and location of households to medical facilities while need factors refer to the health condition such as type and severity of illness (Andersen-Newman, 2005). There is a large volume of literature which indicates that wealth and income affect treatment seeking behaviour especially in accessing formal health facilities (Akin, et al 1995; Gertler and van der Gaag 1990; Sahn et al., 2003).

Insurance in healthcare is an enabling factor in accessing care as it aims to lower prices at the point of care through risk-sharing, thereby improving health outcomes. Literature on health insurance and its effect on treatment-seeking behaviour have been vast and varied. The estimation of this effect is difficult especially in countries where health care is universally provided as there is no possibility to have treatment and control groups following an intervention. In recent times, as many countries encourage the adoption of social health insurance schemes, there are more studies being undertaken in Asia and Sub Saharan Africa (De Allegri, 2008; Ekman, 2007; Hsiao, 2007; Mensah et al., 2010). Chen et al. (2006) found that Taiwan’s NHI greatly increased the utilization of both outpatient and inpatient services. In Ghana, Ansah et al. (2009) in a randomised trial using 2,194 households show that removing out-of-pocket payments for health care had an impact on health care-seeking behaviour. Some of these studies show that people tend to move away from informal or self-medication to a more formal healthcare facilities (Selvaraj, 2012). A study by van den Boom et al. (2008) using the 2005/2006 Ghana Living Standards Survey (GLSS) shows that about 80 percent of NHIS members use government or private hospitals compared to 65 percent of the uninsured.

However, there are inefficiencies generated by an increase in demand for care when patients do not face the full price care often termed moral hazard. This distortion is even deeper as the level and extent of coverage depend on the individual risk aversion, the premium, and the type of insurance contract. While ex ante moral hazard is nearly always mentioned as a potential consequence of health insurance, empirical studies have yet to provide much evidence to support this prediction (Zweifel & Manning, 2000; Kenkel, 2000). Individuals buy health insurance on the basis of several factors. Individuals who are less healthy or suffering from chronic diseases may join the health insurance scheme in order to enjoy its benefits without revealing their true health status. This is often termed as adverse selection and to counteract this, insurers often find possible ways to insure only the healthy (cream skimming). Furthermore, there is also the fact that richer individuals may obtain health insurance for future health benefits (Nyantakyi 2009). Hence these unobservable factors which influence the uptake of health insurance make it difficult to isolate health insurance as the key factor influencing treatment-seeking behaviour (Waters, 1999). Due to inherent biases associated with health status and selecting into health insurance programs, studies using cross-sectional data can only show association among relationships (Roetzheim et al., 2000; Hsia et al., 2000; Merrill, 2001; McWilliams et al., 2004).

## 4. Theoretical Framework and Methodology

### 4.1 Theoretical Model

The theoretical framework is based on the concept of utility maximisation and household production of health similar to that used in past healthcare demand studies (Gertler, et al 1987; Gertler and van der Gaag 1990; Mwabu et al., 1993; Sahn et al., 2003 and Mariko et al, 2003, Abdulraheem, 2007; Nonvignon et al. 2010). This is a direct utility approach where conclusions are drawn based on observations on the individual. The basic assumption is that the rational behaviour of the patient when faced with health seeking decisions will be to choose based on the maximisation of their utility. Individuals must decide between the use and non-use of health services on the first level and secondly make a choice between alternate providers of formal care. During this process, the rational individual aims at maximising utility, which can be expressed by the following function

U_nk_ = U_nk_ (T_k_, P_n_, I_n_, ζ_n_) + ε_nk_ (1)

where T_k_ the travel time to the provider k; P_n_ characteristics of illness of individual n who decides to consult the provider k (these characteristics do not vary by the healthcare choice); I_n_ is the insurance status of individual n and ζ_n_ demographic and socio-economic characteristics of individual n and of his household and ε_nk_ is a vector of all the unobserved components.

Previous authors have used various specifications for demand for healthcare. Since these healthcare decisions are discrete in nature, their estimations can be made using discrete choice formulations. There are three options available and these include multinomial logit (Van den Boom et al., 2008, Kaija & Okwi, 2010), multinomial probit (Akin et al., 1995, Van den Boom et al., 2008; Nonvignon et al., 2010) and nested multinomial logit (Mariko, 2003; Mwabu et al., 1993). This paper adopts the multinomial logit (MNL) model since the dependent variable is unordered and polychotomous. However, this model requires the ‘Indepence of Irrelevant Alternatives’ (IIA) assumption to be satisfied (Nash, 1950; McFadden, 1974). This property requires that the relative probability of choosing between two alternatives is unaffected by the presence of additional alternatives. To check whether this property holds, a Hausman test procedure was run and the results satisfy the assumption (Hausman et al., 1984). Train (2009) however, indicates that the IIA assumption in the MNL model is not as restrictive as it first seems and may be the natural outcome of a well-specified model. The main advantage of the multinomial probit estimator is that it does not need the IIA to be satisfied as it allows more than two courses of action to be estimated within the same equation. However, it requires that all unobserved components to be normally distributed which may not always be the case for certain factors such as price coefficients (Train, 2009). Long and Freese (2006) also point out there is the possibility for errors to be correlated across alternatives in a model fit by mprobit. This would imply the presence of the IIA assumption.

Other econometric considerations as noted earlier include the possible upward biases of some of the explanatory variables if they are found to be correlated with the error term. In addition, the paper aims to understand the extent to wealth influences the effect of health insurance on choice of care by interacting the wealth index with health insurance status. This is entered into the model as a separate explanatory variable. We hypothesis that the effect of having insurance would be less for wealthy households as services would be relatively more affordable to them even without insurance.

Some of the observations involve individuals from the same household and as such errors obtained from these may be correlated with each other. To test the robustness of the estimates, the standard errors produced by the model were corrected for intra-cluster correlation by using the household identification (Wooldridge, 2002). The results are however the same and proves the robustness of the results. STATA 11 was used for all statistical analysis (Stata Corp., 2009).

### 4.2 Sampling and Data Collection

A multi-staged systematic sampling approach to obtain the study population was adopted. Ghana is divided into 10 administrative regions which are subdivided into 170 districts. The administrative districts cut across 3 agro-ecological zones in Ghana namely coastal, forest and savannah. A district was selected in each zone making a total of 3 districts surveyed. A representative household survey was conducted using Enumeration Areas (EAs) based on the 2000 Ghana Population and Housing Census for the selected districts. For each district, 27 EAs, representative of the district were selected. This included both urban and rural communities. Subsequently, 30 households were systematically sampled from the household listing in each EA to obtain the required sample size of 810 in each district; giving a total of 2430 households in all three districts.

The household questionnaire was administered to the head of household between January and April, 2011. For each household, data was collected on individual and household characteristics (income, education, health insurance status, and treatment seeking behaviour, dimensions of quality of care, choice of provider, and reasons for provider choice) as well as community characteristics (whether there was a healthcare facility in the community). Insured members are described as those who have valid health insurance membership cards in the year of the study.

### 4.3 Variables

In the analysis of provider choice, the dependant variable is a polychotomous variable reflecting the four healthcare alternatives: i. Informal care; ii. Regional/district hospitals; iii. Public clinic/health centres/CHPS and iv. Private hospitals/clinics. We consider individuals who reported illness during the last 4 weeks prior to the study. Evidence from the household survey showed that some sought further care elsewhere after remaining unwell but this study was restricted only to where they first sought care. In total, 1,081 individuals within 358 households reported illness in the last 4 weeks, among 11,089 individuals identified within 2430 households. A total of 1,013 reported seeking care and 68 did not make any attempt to seek care.

In this paper, informal care includes all individuals who did not seek care from formal health care providers (regional/district hospitals, public clinic/health centres/CHPS and private hospitals/clinics). These informal sources could include seeking care from a drug store (unlicensed chemical shop) or drug peddler without prescription from authorized medical providers or self-prescribed medication based on self-advice.

**Figure 2:**
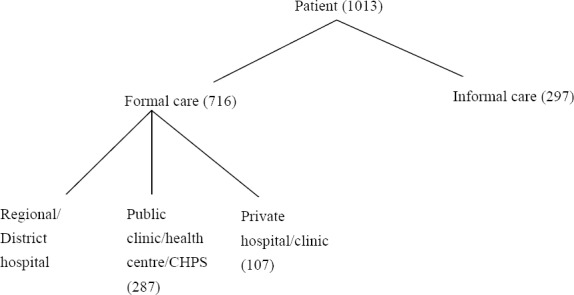
Decision tree showing alternatives of healthcare choice

Among the independent variables are individual, household and community characteristics. Individual characteristics include age, gender, education, health insurance status, nature of illness and travel time to facility (irrespective of mode of transport). Household characteristics include a household welfare index as a proxy for household income. Five variables were created with the fifth quintile (highest income group) used as the base group (the omitted variable). The index was constructed using a collection of durable goods owned by the household, materials used in construction of the home, water and sanitation facilities and size of the home (Rustein et al., 2004). This was calculated using Principal Component Analysis (PCA), a multivariate technique in which a number of related variables are transformed to a set of uncorrelated variables (Montgomery et al., 2000; Jolliffe, 2002; Sahn & Stifel, 2003). The resulting asset scores for households were ordered and used to divide households into quintiles, representing their relative wealth with respect to other households in the study. The wealth index ranged from -1 to 5. The wealth index is particularly valuable in countries like Ghana that lack reliable data on income especially for its large informal sector. We also included a community level characteristic, whether there is a health facility in the community.

## 5. Results

### 5.1 Description of Sample

In total, 11,089 individuals were available for the analysis in the survey data. Of these households, 39 percent were insured and 61 percent uninsured. There were 41 percent rural and 59 percent urban households. The mean household size was 4.6. Table 1 presents the percentage share of individual and household attributes of the insured and non-insured groups. The uninsured had a higher percentage of individuals with no education (38 percent) compared to 28 percent of the insured. We found that among the insured 66 percent lived in urban areas; among the uninsured 55 percent lived in urban areas. Also, among the insured, 28 percent were found to be in the highest wealth quintile compared to 13 percent among the uninsured. In the lowest quintile, we find 12 percent of the insured compared to 25 percent of the uninsured. The results show that households from the fifth quintiles (non poor) were more likely to have valid NHIS card than households belonging to the first quintile (poor). Also, 64 percent of those insured had a health facility in their community whilst 50 percent of the uninsured had a health facility in their community indicating that nearness to a health facility may influence the demand for insurance.

**Table 1:** Socio-demographic characteristics of household members by insurance status (proportions if not indicated otherwise)

Variable	Health Insurance Status			

	Insured		Uninsured	

	Mean	Std. Dev.	Mean	Std. Dev.
**Personal characteristics**				
**Sex**
Female	0.553	0.497	0.494	0.50
**Age**				
Age (mean)	24.692	21.158	22.82	18.686
<18 years	0.498	0.50	0.495	0.50
18-60 years	0.45	0.498	0.482	0.50
≥70 years	0.052	0.223	0.024	0.153
**Education**				
No education	0.284	0.451	0.381	0.486
Some primary	0.383	0.486	0.363	0.481
Completed primary	0.244	0.43	0.21	0.407
Secondary or higher	0.088	0.284	0.046	0.21
**Marital status**				
Never married	0.35	0.477	0.397	0.489
Married/consensual union	0.545	0.498	0.537	0.499
Divorced/separated	0.048	0.213	0.037	0.188
Widowed	0.057	0.232	0.03	0.169
**Residence**				
Urban	0.657	0.475	0.548	0.498
Rural	0.343	0.475	0.451	0.498
**Employment(≥15 years)**				
Formal	0.096	0.294	0.046	0.21
Informal	0.904	0.294	0.954	0.21
**Household characteristics**				
Size (mean)	4.318	2.575	4.768	2.747
Female head	0.244	0.429	0.171	0.377
Age of household head (mean)	50.411	17.205	44.205	15.146
Head with no education	0.364	0.481	0.441	0.497
Head with secondary or higher education	0.144	0.352	0.068	0.252
**Wealth quintiles**				
First (poor)	0.12	0.325	0.25	0.433
Second	0.163	0.369	0.241	0.428
Middle	0.205	0.404	0.197	0.398
Fourth	0.229	0.42	0.185	0.389
Fifth (non-poor)	0.283	0.45	0.127	0.333
**Community**
Health facility in community	0.641	0.479	0.498	0.5

Source: Household data January to April, 2011.

### 5.2 Utilization of Health Services

In this section we present a summary of the choice of provider by wealth quintiles, gender, health insurance status, age, settlement type, educational level and nature of illness (Table 2). The *P*-values of the differences in the categories is reported in column 6 of Table 2. In total, 1013 of those who reported illness in the last 4 weeks sought some form of care. Out of those who sought care in the previous 4 weeks, 60 percent were insured and 40 percent uninsured. Of the total, 71 percent of them sought care from a formal health facility with 72 percent of them insured and 28 percent uninsured. Among the insured, 40 percent consulted the regional/district hospital, followed by 32 percent who chose health centre/clinic and 14 percent chose private hospital/ clinic whilst 14 percent chose informal care. In the uninsured group, 20 percent consulted the regional/district hospital, 23 percent chose health centre/clinic, 6 percent chose private hospital/ clinic whilst 51 percent chose informal care.

**Table 2:** Choice of care among individuals seeking care by insurance status, demographic and socio-economic characteristics and type of illness

Variable	Regional/District hospital (%)	Private hospital/clinic (%)	Public health centre/clinic (%)	Informal Care (%)	P-value*
**Insurance Status**	N=319 **(31.7)**	N=106 **(10.5)**	N=286 **(28.4)**	N=295 **(29.3)**	
**Insured**	39.9	13.6	32.1	14.4	P= 0.000
**Uninsured**	19.8	6.1	22.7	51.3	
**Wealth quintiles**					
**First (poor)**	21.1	11.5	31.3	36.1	P= 0.006
**Second**	26.5	10.5	29.7	33.3	
**Third**	37.0	7.9	26.9	28.0	
**Fourth**	31.7	11.2	26.3	30.7	
**Fifth (non poor)**	40.2	11.5	27.8	20.5	
**Sex**					
**Male**	30.2	9.3	30.4	30.2	P= 0.377
**Female**	32.9	11.5	26.8	28.7	
**Age**					
**<18**	24.5	10.5	34.6	30.4	P= 0.000
**18-69**	36.5	10.1	23.2	30.2	
**70+**	41.2	12.8	25.5	20.6	
**Education**					
**No education**	32.2	11.3	21.6	35	P = 0.034
**Some primary**	31	7	34.7	27.3	
Middle/JSS**	36.4	10	28.2	25.5	
**Secondary & above**	32.9	10.5	26.3	30.3	
**Residence**					
**Urban**	36.5	11.1	26.1	26.4	P = 0.000
**Rural**	24.3	9.7	31.9	34	
**Illness type**					
**Malaria/Fever**	24.2	11.7	35.2	28.9	P = 0.000
**Other Acute diseases**	33.3	8.6	25.5	32.7	
**Chronic diseases**	44.2	12.8	22.4	20.5	

* Chi-square test.

** Juniour Secondary School.

Source: Household data January to April, 2011.

These results were significant at 1% level, showing distinctly that a larger share of the uninsured individuals chose informal care than the insured when seeking care. There is not much variation in choice of health provider by education level for the formal facilities but 35 percent of those with no education chose informal care compared to 30 percent of those with secondary or above. Of those who reported suffering from malaria, the highest percentage (35 percent) chose public health centre/clinic followed by 29 percent who opted for informal care. Fewer individuals with chronic diseases in the sample choose informal care. Of those who reported chronic problems only 20 percent chose informal care.

**Figure 3:**
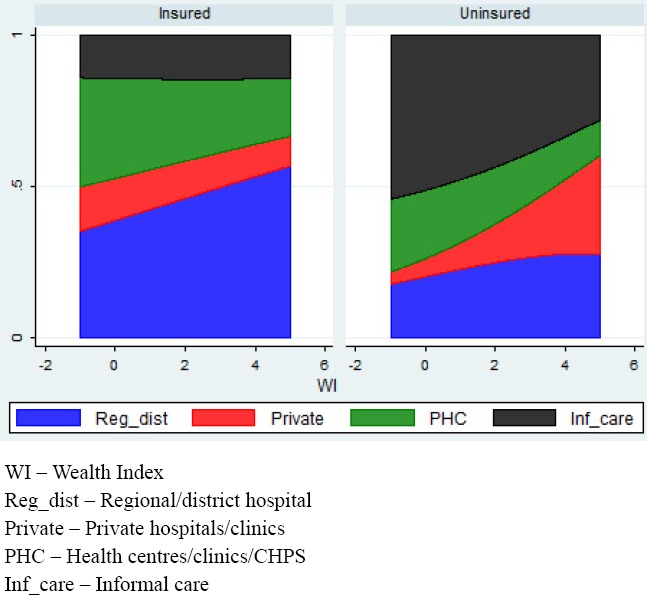
Choice of Care among Individuals Seeking Care in the Past 4 Weeks by Wealth Index and Health Insurance Status among insured and uninsured members

Figure 3 illustrates the choice of care by insurance status. About 85.6 percent of insured individuals who reported an illness episode during the recall period visited a health facility, compared with only 48.7 percent of uninsured individuals. This is statistically significant at 1% level. As indicated by the results, a higher proportion of the insured sought care at the regional/district hospitals followed by public health centres and then private health facilities.

We also see that as wealth increases, the choice patterns change in both the insured and uninsured groups. In the insured group, the choice of a regional/district hospital becomes the preference as wealth increases. In the uninsured group we see a similar trend in the choice of care from private hospitals/clinics indicating that the wealthier uninsured members are more likely to seek care from private hospitals/clinics or regional/district hospitals and less likely to seek informal care.

### 5.3 Main Reasons for Choice of Healthcare

Individuals were asked to give one main reason influencing their choice of a healthcare provider. Results presented in Table 3 indicate that of the total sample, proximity to facility was the most frequent reason followed by good quality care. For those who chose informal care almost 50 percent gave proximity as the main reason whilst 10 percent indicated low charges. Over 60 percent of those who chose regional/district hospitals and private hospitals gave good quality care as their main reason for choice. About 65 percent of those who chose health centres/clinics and CHPS indicated that proximity was the main reason of choice. A comparison by health insurance status shows that 51 percent of the uninsured were influenced by proximity to source of care followed by 27 percent citing good quality care and 5 percent being influenced by low charges. For the insured group, 47 percent cited good quality care, 41 percent were influenced by proximity and about 2 percent influenced by low charges (Table 4). These differences are statistically significant at 1% level.

**Table 3:** Major reason for choice of care by healthcare facility

	Total N	Proximity %	NHIS provider %	Good quality care %	Low charges %	Other %
**Regional/district hospitals**	322	28.9	2.5	63.4	0	5.3
**Private hospital/clinics**	107	28	0.9	60.8	1.9	8.4
**Public health centre/clinic/CHPS**	287	65.5	0.7	26.5	0.4	7
**Informal Care**	295	49.2	0	13.9	10.2	26.8
**Total**	1011	45.1	1.1	38.2	3.3	12.4

*p* = 0.000 χ^2^= 318.707.

Source: Household data January to April, 2011.

**Table 4:** Major Reason of Choice of Care by Health Insurance Status

	Total N	Proximity %	NHIS provider %	Good quality care %	Low charges %	Other %
**Insured**	604	41.2	1.8	46.7	1.8	8.4
**Uninsured**	407	51.1	0	26.5	5.4	17
**Total**	1011	45.2	1.1	38.6	3.3	11.9

*p* = 0.000 χ^2^= 72.426.

Source: Household data, January to April, 2011.

### 5.4 Results of the Multivariate Analysis

This section presents the results from the MNL estimation using 4 models with choice of informal care serving as the reference group in each case. A description of the variables and their summary statistics is shown in Table A in the Appendix.

#### 5.4.1 Description of Models

In order to evaluate the effect of health insurance and wealth, we create four different models. In Model 1, we look at the effect of wealth index alone omitting the health insurance variable. In Model 2 we include the health insurance variable and a multiplicative variable between health insurance and wealth index (INSUR*WI), to take into account the fact that health insurance may vary with wealth. In Model 3, in addition to the health insurance variable we create quintile categories and use the highest quintile dummy as the reference category. The quintile categories are WQ_1, WQ_2, WQ_3, WQ_4 and WQ_5. Model 4 is similar to Model 3 but the difference is we add a multiplicative between insurance and the quintiles of wealth, to evaluate the statistical significance of the interaction between health insurance and the various wealth categories. In all the models we control for age, gender and education. The results are presented in Tables 6, 7, 8 and 9.

**Table 5:** Relative Risk Ratios Estimation of Model (1) showing the Probability of Choice of Healthcare in last 4 weeks, omitting the Health Insurance Variable

	Regional/district	Private hospital/clinic	Public health centre
**WEALTH INDEX**	1.549***	1.326*	1.138
	[0.17]	[0.20]	[0.13]
**PRIMARY EDUC**	1.375	0.767	1.827**
	[0.35]	[0.29]	[0.46]
**JSS EDUC**	1.057	0.799	1.669*
	[0.28]	[0.30]	[0.46]
**SECONDARY EDUC**	0.543	0.523	1.159
	[0.21]	[0.29]	[0.45]
**MALE**	0.990	0.868	1.044
	[0.20]	[0.25]	[0.21]
**MALARIA/FEVER**	0.676	0.992	1.295
	[0.22]	[0.42]	[0.43]
**OTHER ACUTE**	0.642	0.501*	0.753
	[0.18]	[0.19]	[0.23]
**HFAC IN COMMUNITY**	1.674**	1.785	1.418
	[0.37]	[0.56]	[0.31]
**< 18 YEARS**	0.630*	0.742	1.174
	[0.17]	[0.28]	[0.30]
**> 70 YEARS**	1.600	1.676	1.882*
	[0.55]	[0.75]	[0.68]
**TIME TO FACILITY**	1.042***	1.042***	1.031***
	[0.01]	[0.01]	[0.01]

Robust standard errors are in parentheses.

* Significant at 10%.

** Significant at 5%.

*** Significant at 1%.

Source: Household data January to April, 2011

**Table 6:** Relative Risk Ratios Estimation of Model (2) showing the Probability of Choice of Healthcare in last 4 weeks, with INSUR*WI interaction

	Regional/district hospital	Private hospital/clinic	Public health centre/CHPS
**HEALTH INSURANCE**	6.569***	11.005***	6.205***
	[1.48]	[3.98]	[1.39]
**WEALTH INDEX**	1.633**	1.315	1.036
	[0.30]	[0.46]	[0.21]
INSUR*WI	0.752	0.788	0.893
	[0.16]	[0.30]	[0.21]
**PRIMARY EDUC**	1.395	0.779	1.882**
	[0.38]	[0.30]	[0.51]
**JSS EDUC**	1.039	0.774	1.675*
	[0.30]	[0.31]	[0.49]
**SECONDARY EDUC**	0.398**	0.371*	0.841
	[0.16]	[0.21]	[0.34]
**MALE**	1.179	1.044	1.234
	[0.26]	[0.47]	[0.26]
**MALARIA/FEVER**	0.930	1.421	1.801*
	[0.32]	[0.63]	[0.64]
**OTHER ACUTE**	0.875	0.721	1.022
	[0.26]	[0.29]	[0.33]
**HFAC IN COMMUNITY**	1.377	1.429	1.187
	[0.33]	[0.47]	[0.28]
**< 18 YEARS**	0.540**	0.643	1.014
	[0.15]	[0.25]	[0.28]
**> 70 YEARS**	1.008	0.986	1.218
	[0.37]	[0.48]	[0.46]
**TIME TO FACILITY**	1.039***	1.040***	1.028***
	[0.01]	[0.01]	[0.01]

Robust standard errors are in parentheses.

* Significant at 10%.

** Significant at 5%.

*** Significant at 1%.

Source: Household data January to April, 2011.

**Table 7:** Relative Risk Ratios Estimation of Model (3) showing the Probability of Choice of Healthcare in last 4 weeks, with wealth quintiles

	Regional/district hospital	Private hospital/clinic	Public health centre/CHPS
**HEALTH INSURANCE**	6.551***	12.051***	6.503***
	[1.48]	[4.39]	[1.467]
**WQ_1**	0.263***	0.807	1.087
	[0.11]	[0.42]	[0.41]
**WQ_2**	0.524*	1.074	1.067
	[0.18]	[0.51]	[0.36]
**WQ_3**	0.628	0.534	0.864
	[0.21]	[0.25]	[0.29]
**WQ_4**	0.474**	0.558	0.556*
	[0.14]	[0.24]	[0.17]
**PRIMARY EDUC**	1.425	0.828	1.987**
	[0.11]	[0.32]	[0.54]
**JSS EDUC**	1.052	0.799	1.752*
	[0.30]	[0.32]	[0.52]
**SECONDARY EDUC**	0.397**	0.370	0.859
	[0.16]	[0.21]	[0.35]
**MALE**	1.174	1.073	1.237
	[0.26]	[0.32]	[0.27]
**MALARIA/FEVER**	0.958	1.473	1.843*
	[0.33]	[0.65]	[0.65]
**OTHER ACUTE**	0.882	0.699	1.002
	[0.27]	[0.28]	[0.32]
**HFAC IN COMMUNITY**	1.375	1.681	1.294
	[0.33]	[0.58]	[0.31]
**< 18 YEARS**	0.534**	0.607	0.987
	[0.15]	[0.24]	[0.27]
**> 70 YEARS**	1.017	0.929	1.207
	[0.37]	[0.44]	[0.46]
**TIME TO FACILITY**	1.039***	1.040***	1.027***
	[0.01]	[0.01]	[0.01]

Robust standard errors are in parentheses.

* Significant at 10%.

** Significant at 5%.

*** Significant at 1%.

Source: Household data January to April, 2011

**Table 8:** Relative Risk Ratios Estimation of Model (4) showing the Probability of Choice of Healthcare in last 4 weeks, with Wealth Quintiles and Insurance Interaction

	Regional/district hospital	Private hospital/clinic	Public health centre/CHPS
**HEALTH INSURANCE**	5.257***	8.667**	3.903***
	[2.48]	[7.22]	[1.885]
**WQ_1**	0.267**	0.359	1.024
	[0.16]	[0.39]	[0.52]
**WQ_2**	0.410*	1.153	0.582
	[0.21]	[1.03]	[0.29]
**WQ_3**	0.698	0.287	0.651
	[0.35]	[0.37]	[0.34]
**WQ_4**	0.346*	0.499	0.275**
	[0.19]	[0.54]	[0.17]
INSUR*WQ_1	0.866	2.410	0.887
	[0.67]	[2.96]	[0.63]
INSUR*WQ_2	1.963	1.190	3.191*
	[1.33]	[1-24]	[2.16]
INSUR*WQ_3	0.866	2.011	1.430
	[0.57]	[2.79]	[0.97]
INSUR*WQ_4	1.762	1.385	2.822
	[1.19]	[1.64]	[2.04]
**PRIMARY EDUC**	1.476	0.823	2.110***
	[0.41]	[0.33]	[0.54]
**JSS EDUC**	1.073	0.796	1.827**
	[0.31]	[0.32]	[0.55]
**SECONDARY EDUC**	0.388**	0.366	0.841
	[0.16]	[0.21]	[0.35]
**MALE**	1.180	1.092	1.245
	[0.26]	[0.34]	[0.27]
**MALARIA/FEVER**	0.939	1.469	1.818*
	[0.33]	[0.65]	[0.65]
**OTHER ACUTE**	0.838	0.689	0.944
	[0.26]	[0.28]	[0.31]
**HFAC IN COMMUNITY**	1.447	1.724	1.374
	[0.36]	[0.59]	[0.33]
**< 18 YEARS**	0.514**	0.599	0.940
	[0.15]	[0.24]	[0.26]
**> 70 YEARS**	0,989	0.922	1.161
	[0.36]	[0.44]	[0.45]
**TIME TO FACILITY**	1.039***	1.040***	1.027***
	[0.01]	[0.01]	[0.01]

Robust standard errors are in parentheses.

* Significant at 10%.

** Significant at 5%.

*** Significant at 1%.

Source: Household data January to April, 2011.

We present the relative risk ratios (RRR) for each type of health facility chosen. The RRR is interpreted as the relative probability of choosing alternative *k* to informal care (the comparison group for the MNL estimation) for individuals with a particular characteristic, compared to the comparison group. For each of the independent variables, the comparison groups have been indicated in Table A in the Appendix.

#### 5.4.2 Determinants of Treatment Choice

The findings show that health insurance is a significant determinant of choice of care. This is indicated in models 2, 3 and 4. For instance those insured are 6 times more likely to choose regional/district hospitals and health centres/clinics and 11 times more likely to choose private hospitals/clinics over informal care when compared to those uninsured and this is significant at 1% (Table 7).

Wealth index variable shows significance only with the choice of regional/district hospitals (1% significance level). The relative risk ratio for one-unit increase in wealth increases the probability of choosing regional/district hospitals over informal care. This implies that the wealthier you are the more likely seek care from more expensive medical care. Using the wealth quintiles, the findings show that compared to the fifth quintile (non poor), those in the first quintile (poor) are less likely to choose regional/district hospitals (1% significant level). The same applies to those in the fourth quintile but at 5% significance level. There is no significant difference between quintiles in the other choice options. However, the results imply that those with fewer assets are more likely to rely on informal care as a cheaper alternative to formal care. Results with the multiplicative term presented in Models 2 and 4 show no significant effect. The interaction terms between health insurance and the wealth quintiles are not significant for any of the quintiles. This may indicate that wealth status does not influence the effect of health insurance on choice of care as hypothesised.

Age, gender, nature of illness and having a health facility in community are not significant determinants of choice. However, the results indicate that individuals with self-reported malaria/fever are 1.8 more times likely to choose public health centres/clinics over informal care, when compared to those with chronic diseases and this is significant at 10%.

The education variable is not significant with the choice of care from the regional/district hospitals as well as private hospitals/clinics but highly significant in the choice of health centres/clinics. For instance in Model 4 individuals with primary education are 2 times more likely to choose public health centres/clinics over informal care, when compared to those with no education. This is significant at1% and similar results are reported in Models 1, 2 and 3 at 5% significance level. This finding could imply that those with schooling up to primary school level may be better informed about the health risks of using informal care than those with no education with regards to choosing public health centres/clinics. However, the results also show that individuals with secondary or above level of education are 0.4 times less likely to choose regional/district hospitals over informal care, when compared to those with no education (significant at 5%). This is the case with all models with exception of Model 1. Perhaps, indicating that the- most-educated face a greater opportunity cost of their time compared to those with no education.

Travel time to facility irrespective of mode of transportation is a significant determinant of choice of care (1% significance level) for all the different choices of health facilities relative to informal care. This is indicated in all the 4 models. However the relative risk of 1 and for the exposure status to be related to the outcome, the relative risk must differ from 1. The results from the estimation implies that there is only a small difference in the way a unit increment in travel time (measured in minutes) affects the two groups.

## 6. Discussion

This paper seeks to investigate factors that affect the treatment-seeking behaviour of individuals when ill in Ghana and establish any relevant differences between those insure and the uninsured. Noticeably, health insurance status and travel time to facility have been shown to be the two main determinants of healthcare demand. Although significant, there is a small difference between the effect of travel time in the two groups. These main factors influencing treatment-seeking behaviour are largely related to the enabling factors rather than predisposed factors such as age, gender or educational status. The link between insurance and choice of provider is quite evident in the clear differences exhibited by the two groups. A large proportion of the insured who reported ill, sought care from formal health care providers compared to the uninsured which confirms our original hypothesis. A number of previous studies illustrated in the review section also point to this feature. Given that one of the main aims of the NHIS scheme is to improve access to healthcare this could be an early indication of the success of this policy intervention. Quite clearly having a valid health insurance encourages members to seek care from formal healthcare facilities.

However, looking beyond this sub sample of those seeking treatment, we reflect on the fact that 61 percent of the total sample population in this study do not have valid NHIS cards or have never insured under the scheme. Although we did not seek to understand why individuals were not enrolled in the scheme, one of the enabling factors we flagged was wealth status. Also included was an interactive term between wealth and health insurance, to ascertain the effect of wealth status on the choice of care as a result of health insurance. The effect of this interaction term was not significant; however, our results indicate that health insurance status was lower among the poorer quintiles. Previous studies in Ghana have also shown that individuals from richer quintiles are more likely to be enrolled into the NHIS scheme than those in poorer quintiles (Asante & Aikins, 2008; Bjerrum & Asante, 2009; Chankova, Atim, & Hatt, 2009). Mou et al. (2009) also find health insurance lowest in the vulnerable groups. Embedded in the NHIS Act is an exemption policy which when implemented effectively will ensure proper identification of poor and vulnerable groups who need to be financially assisted to register and become members of the scheme. For the scheme to maintain its relevance as a tool to promote universal coverage there is the need to develop the requisite strategies to reduce the barriers that discourage poor and vulnerable groups from enrolling in the scheme.

We also find significant differences in treatment choices between the insured and uninsured members. The results show that a higher proportion of the insured sought care at the regional/district hospitals followed by public health centres and then private health facilities. In the uninsured group, a higher proportion sought care at the public health centres followed by regional/district hospitals and then private health facilities. Further analysis reveal, that a higher proportion of the insured emphasised that quality of care influenced their choice of provider whilst a higher proportion of the uninsured indicated that proximity influenced their choice of care. Over 63 percent of those who chose regional/district hospitals cited good quality care as their main reason for choice whilst 66 percent of those opting for public health centres cited proximity as their main reason for choice.

Given the hierarchical organisation of the health sector, it is expected that public health centres would be the first point of care since they are geographically closer to most households. However, the findings in this paper indicate that the about double the number of the insured sought treatment at regional/district hospitals compared to the uninsured. It is not definite whether the insured are bypassing lower level facilities for higher level facilities. Additional we cannot conclude that the uninsured are choosing proximity over good quality because of financial barriers. We however agree that the uninsured face higher cost barriers than the insured. Since this study did not expand the predictive factors to include quality of care, the effect of quality of care indicators is unknown and remains a limitation in this study. We are also cautious given the cross-sectional nature of the study to claim any causal effect of health insurance on treatment seeking-behaviour.

## 7. Conclusion and Policy Implications

Understanding patient choice of healthcare provider in a health care delivery system is of considerable policy significance. We analyze the treatment-seeking behaviour of individuals reporting illness in the framework of the NHIS in Ghana. The results indicate that health insurance is a significant determinant of choice of provider. Compared to the uninsured, the insured are more likely to choose formal health facilities than informal care which confirms our initial hypothesis and also the results of other studies conducted on the NHIS in Ghana. A conclusion which is underscored in many relevant studies on the effect of expanding health insurance programmes in many of the low to middle income countries that are adopting various degrees of social health insurance programs.

In a nutshell, our study confirms the positive impact of the NHIS scheme in lowering the financial burden of the health care cost to households who are able to pay or are subsidised to pay the health insurance premium. Yet the study reveals also the regressive nature of the scheme particularly to poorer households. Equity concerns about the NHI scheme have been raised in various quarters. It still seems that vulnerable groups are suffering the same fate as they did in the ‘Cash and Carry’ system. Ensuring equitable access for vulnerable groups is important and the exemption policy which forms part of the NHIS scheme must be effectively implemented. In spite of these shortcomings, we agree that the implementation of Ghana’s NHIS falls in line with some of the strategies outlined for countries to attain

## Appendix

**Table 1:** Description of variables in estimation

Dependant variable	Variable Abbreviation	Mean	Std. Dev.	N
Regional/district hospital		0.318	0.466	1013
Private hospital/clinic		0.106	0.308	1013
Public health centre/clinic		0.283	0.451	1013
Informal care*		0.293	0.455	1013

**Independent variables**				
***Individual characteristics***				
age=<18 years	< 18 YEARS	0.432	0.495	1081
18-69*		0.462	0.499	1081
70 years and above	> 70 YEARS	0.101	0.301	1081
Male	MALE	0.421	0.494	1081
Female*		0.579	0.494	1081
No education*	NO EDUC	0.36	0.48	950
Some primary	PRIMARY EDUC	0.314	0.464	950
JSS/Middle	JSS EDUC	0.244	0.429	950
Secondary and above	SECONDARY EDUC	0.082	0.274	950
Insured	HEALTH INSURANCE	0.585	0.493	1081
Uninsured*		0.415	0.493	1081
Malaria/fever	MALARIA/FEVER	0.342	0.475	1067
Other acute	OTHER ACUTE	0.499	0.5	1067
Chronic*	CHRONIC	0.159	0.366	1067

**Household characteristics:**				
Wealth Quintile				
First	WQ_1	0.181	0.385	1081
Second	WQ_2	0.206	0.404	1081
Middle	WQ_3	0.184	0.388	1081
Fourth	WQ_4	0.198	0.198	1081
Fifth*	WQ_5	0.229	0.229	1081

**Community characteristics**				
Health facility in Community	HFAC IN COMMUNITY	0.584	0.493	1081
Health facility not in Community*		0.416	0.493	1081
Travel time to facility**		27.559	43.068	965

* Comparison group.

** travel time in minutes.

Source: Household data January to April, 2011.
